# Durability of Osteo Fillings

**Published:** 1899-07

**Authors:** 


### Durability of Osteo Fillings.

The durability of an osteo filling is greatly increased by condensing it thoroughly by pressure while it is setting. This is best done with a smooth, round-headed hand burnisher, kneading it, so to speak, into the cavity. The rubber dam should be used, and as much care taken in shaping and finishing the cavity as for a gold filling. A stopping so treated with melted paraffin flowed over, using the hot-air syringe to keep it melted while it soaks into the cement will be found (the conditions of the mouth being favorable) to last for many years, even in the most exposed positions. It is as remarkable as it is true that the walls of carefully prepared cavities that have been filled with osteo seldom show any signs of reoccurring decay, although the stopping may be worn away considerably below the enamel edge.—*Pharmaceutical Journal*

